# A Longitudinal Examination of Military Veterans’ Invictus Games Stress Experiences

**DOI:** 10.3389/fpsyg.2019.01934

**Published:** 2019-08-22

**Authors:** Gareth A. Roberts, Rachel Arnold, James E. Turner, Martin Colclough, James Bilzon

**Affiliations:** ^1^Department for Health, University of Bath, Bath, United Kingdom; ^2^Help for Heroes Sports Recovery, Tidworth, United Kingdom

**Keywords:** competition, cortisol, emotion, psychological stress, rehabilitation, sport

## Abstract

This study explored patterns of change in stress variables (i.e., stressors, appraisals, emotions) encountered by wounded, injured, and sick military veterans in the build up to, during, and following an international sporting competition. The study also examined interactions between psychosocial variables and salivary biomarkers of stress and how these relate to veterans’ health, well-being, illness, and performance. 40 Invictus Games (IG) athletes and a control group of 20 military veteran athletes completed questionnaires at seven time points over a 12-week period. Furthermore, participants provided morning and evening saliva samples at four time points to measure cortisol and secretory immunoglobulin A. Multilevel growth curve analyses revealed significant changes in growth trajectories of stress-related variables. For example, team and culture stressors and anger and dejection emotions significantly increased in the build up to competition, whilst challenge appraisals and excitement and happiness emotions significantly decreased over the same time-frame. A number of the stress related variables also predicted performance, well-being, and mental health. Specifically, organizational stressors and threat appraisals were found to negatively relate to performance, well-being, and mental health. Furthermore, whilst challenge appraisals and problem focused coping positively related to veterans’ well-being, adopting emotion-focused and avoidance coping strategies negatively predicted well-being and mental health. Turning to emotions, experiencing anger, anxiety, and dejection negatively related to mental health, well-being and performance; whereas happiness and excitement displayed a positive relationship with these outcomes. The findings also highlighted that organizational stressor intensity was positively related to cortisol exposure at competition. To conclude, this study not only provides a novel, longitudinal, interdisciplinary insight into psychological and biological markers of the stress response as it relates to the performance, health, and well-being of military veterans, but also further contributes to theoretical understanding on the transactional nature of stress. Moreover, the findings significantly contribute to practice regarding how best to support this unique population in adaptively responding to and engaging with competitive sport.

## Introduction

Research conducted with military veterans illustrates that sport can provide significant physiological, psychological, and social benefits for recovery (Sporner et al., 2009; Caddick and Smith, 2014). To achieve these benefits, the Invictus Games (IG) were created to offer a large number of wounded, injured, or sick Armed Forces personnel and veterans the opportunity to compete in an international sport competition. Despite participation benefits, operating in such a demanding sporting environment can also produce undesirable outcomes (e.g., unpleasant emotions, performance dissatisfaction; Nicholls et al., 2012). Therefore, it is important to examine veterans’ holistic experiences of high-level sport and the antecedents to both positive and negative outcomes, which have not been studied to date. Furthermore, the majority of research conducted on stress in sport, has been cross-sectional; thus inhibiting causal inferences and not accurately reflecting the dynamic nature of the stress process. As such, a transactional approach (cf. Lazarus and Folkman, 1984) can be adopted to underpin such enquiries. The transactional stress theory suggests that stressors arise from the environment the performer operates in, are mediated by the processes of perception, appraisal, and coping, and, as a consequence, result in positive or negative responses, feeling states, and outcomes (Fletcher et al., 2006, p. 333).

In line with transactional stress theory, environmental demands are often examined as the first component of the process, as they can indicate triggers of certain responses. Furthermore, in early definitions of stress (i.e., stimulus based; see Fletcher et al., 2006), the environmental conditions faced by individuals are emphasized. Research has identified three types of demands: competitive, organizational, and personal; with organizational-related demands found to be experienced and recalled more often than competitive-related demands (Hanton et al., 2005). Within sport, organizational stressors are defined as “the environmental demands associated primarily and directly with the organization within which an individual is operating” (Fletcher et al., 2006, p. 329). Organizational stressors can be prevalent and problematic for a range of sport performers who compete at various competitive levels (Arnold and Fletcher, 2012a; Fletcher et al., 2012a; Arnold et al., 2016a, b). Specifically, Arnold et al. (2016b) sampled elite athletes with a disability and identified 316 organizational stressors which were categorized into 31 concepts and four, previously conceptualized dimensions: leadership and personal issues (e.g., the coach’s behavior and interactions), cultural and team issues (e.g., teammate’s personality and attitudes), logistical and environmental issues (e.g., rules and regulations), and performance and personal issues (e.g., transitions). Further to this, organizational stressors have been linked to various outcomes including, emotions, motivation, well-being, performance, and burnout (Fletcher et al., 2012b; Tabei et al., 2012; Larner et al., 2016; Arnold et al., 2017, 2018; Bartholomew et al., 2017; Wagstaff et al., 2018). The samples recruited for this research on outcomes of organizational stressors, however, has typically been able-bodied sport performers with little attention afforded to the experiences of disabled sport performers. Furthermore, when examining the stress experienced by military veterans, the focus to date has tended to be on reported outcomes including post-traumatic stress disorder (PTSD), depression, and alcohol abuse (Fear et al., 2010; Murphy et al., 2016). Notwithstanding the importance of examining such consequences, research has suggested that veterans can encounter stressors directly associated with supporting organizations (cf. Weir et al., 2017), though this has been afforded limited attention to date. Organizational stressors are of particular interest in the current study considering the affiliation of the United Kingdom IG team to the military charity, Help for Heroes, whose Sports Recovery (HfHSR) team’s mission is to support athletes pre, during, and post-competition.

After encountering a demand, such as the aforementioned organizational stressors, individuals make a cognitive evaluation on its meaning and significance in relation to their beliefs, values, goal commitments, and situational intentions (Lazarus and Folkman, 1984). This primary appraisal is informed by the individual’s initial perception of whether a stressor is irrelevant, benign-positive, or stressful. A stressful encounter occurs when the situation is evaluated as significant to an individual’s well-being and, subsequently, there are three possible appraisals: harm/loss, threat, or challenge (Fletcher et al., 2006). These cognitive appraisals can then determine whether individuals respond adaptively or maladaptively to motivated performance situations (cf. Blascovich, 2008), with research suggesting that challenge appraisals are considered more beneficial to performance than threat appraisals (Nicholls et al., 2011). If something is considered at stake, individuals will engage in secondary appraisal to evaluate the availability of coping resources (Lazarus, 2000). Research suggests that organizational stressors are predominantly appraised as harmful, with little perceived control, and few coping resources available (Didymus and Fletcher, 2014). In military settings, evidence suggests that appraisals are key, alongside coping strategies, to develop positive, mental health outcomes (Solomon et al., 1988). No research to date, however, has examined the appraisals made by military veterans during their sporting involvement.

According to the transactional theory of stress, emotions arise following the cognitive appraisal of a situation (Lazarus, 2000). In the sport setting, research has found associations between threat appraisals and the generation of unpleasant emotions (e.g., anger, anxiety), and between challenge appraisals and positive emotions (e.g., happiness, excitement) (Nicholls et al., 2011). The aforementioned emotions have also been found to be common responses to organizational stressors (Fletcher et al., 2012b). It is important to note, however, that given the lack of research conducted with military veterans, as well as the nature of military veterans’ previous occupation and experience, it may be erroneous to assume that the patterns of stress, appraisal, and emotions will be similar to those observed in other populations (cf. Fletcher and Arnold, 2017). Additionally, the transactional stress process can be moderated by various personal and situational characteristics (Fletcher et al., 2006) which are yet to be explored with a military veteran population. When examining emotions in the military veteran population, exercise rehabilitation programs have been shown to help reduce negatively valenced emotions, as well as improve mood states (Otter and Currie, 2004). Literature could be advanced, however, to ascertain whether the same longitudinal effect exists for veterans who compete in sport. Turning to coping, sport research indicates that individuals should engage in task-orientated coping strategies in order to perform maximally, generate positive emotions, and improve physical and mental health (Nicholls et al., 2012). There is a need to examine, however, the ways in which veterans cope with sporting pressures, since the focus of military research to date has been around coping on a mission or within military occupations (Barnett et al., 2016).

Turning to the final component of the transactional stress theory, it would be beneficial to examine the impact of veterans’ stress on psychological, behavioral, and immune and endocrine measurements. Research to date has investigated the impact of organizational stressors on psychosocial outcomes (e.g., performance, well-being) in a cross-sectional manner (see, for a review, Fletcher and Arnold, 2017), yet has not examined this longitudinally, nor their impact on immune and endocrine function. Chronic psychological stress can impair aspects of immune function, potentially increasing the chance of developing infections (Segerstrom and Miller, 2004; Pedersen et al., 2010). To explain this link, psychological stress influences immune function via alterations to the sympathetic-adrenal-medullary axis and the hypothalamic pituitary adrenal axis (Ader et al., 1995). Changes include abnormal sympathetic and parasympathetic stimulation of the immune system and alterations to the diurnal rhythm of the endocrine system; thus, changing overall exposure to hormones such as cortisol, which can impair immune function (Ader et al., 1995). Evidence shows that elite athletes and para-athletes report a high frequency of illness symptoms around the time of mass-participation sporting events (Derman et al., 2014; Bonini et al., 2015). A frequently cited explanation is that high volumes and intensities of exercise might impair aspects of immune function, increasing infection risk (Campbell and Turner, 2018). There is limited evidence to support this idea, and a more likely explanation is increased exposure to infections due to crowds (Choudhry et al., 2006; Campbell and Turner, 2018). If immunological alterations are evident, environmental stressors (e.g., sleep disruption, international flights), or indeed, psychological stress are most likely to be the factors affecting immune function (Taylor et al., 2015; Campbell and Turner, 2018). Thus, if psychological stress in the lead up to a sporting competition impairs immune function, then the chance of developing an infection due to attending a crowded mass-participation event could be exacerbated. One immune component that might be affected is secretory immunoglobulin-A (S-IgA), which provides a first line of defense against infections on mucosal surfaces (e.g., the lining of the mouth, nose, and airways). Previous studies have reported an inverse relationship between salivary S-IgA and symptoms of upper respiratory tract infections (URTIs; Neville et al., 2008; Mortatti et al., 2012) which coincide with increased levels of salivary cortisol (Cunniffe et al., 2011; Casto and Edwards, 2016) in athletes (ranging from novice to elite) at competition. It is often not examined in studies, however, whether psychological stress is driving immune function changes.

Based on the above review of extant literature, the primary purpose of this study is to examine the stress experiences of veterans in preparation for, during, and post the IG and to quantify how these change over time and in comparison to a control group not participating in high-level competition. A secondary purpose is to examine the relationships between stress (e.g., stressors, responses) and psychological, behavioral, and immune and endocrine measurements. In line with this latter outcome, a third purpose is to examine whether psychological stress is associated with changes in biomarkers of stress (i.e., salivary cortisol) potentially impacting immune function (i.e., salivary S-IgA) and symptoms of URTI.

## Materials and Methods

### Participants

Forty participants were recruited from the 2016 United Kingdom IG Team (29 males, 11 females) who ranged in age from 24 to 51 years (*M*_*age*_ = 37.4 ± 8.6 years), and had served in either the British Army (*n* = 27), Royal Navy (*n* = 2), Royal Marines (*n* = 3), or Royal Air Force (*n* = 8) for an average of 12.7 ± 7.0 years. The IG group identified themselves as having various mental health issues (*n* = 3), or physical (*n* = 32), hearing (*n* = 1), visual (*n* = 1), or cognitive impairments (*n* = 1), or other injuries (*n* = 2); and reported that they had their injury/impairment for an average of 6.6 ± 5.2 years. Participants were competing at the Games in eight sports (e.g., Archery, Rowing, Powerlifting, Cycling, Swimming, Athletics, Wheelchair Basketball, and Wheelchair Rugby), with some veterans having never previously competed in their sport (*n* = 14), whereas others had competed from 3 months to 17 years (*M*_*years*_ = 4.6 ± 6.4), at standards ranging from club to international level.

Twenty military veterans who did not participate in the Games but still engaged in competitive sport were recruited as a control (CON) group (16 males, 4 females) who ranged in age from 24 to 62 (*M*_*age*_ = 42.5 ± 11.4). The CON group had served in either the British Army (*n* = 15), Royal Marines (*n* = 2), Royal Air Force (*n* = 2), or Royal Navy (*n* = 1) for an average of 15.5 ± 11.0 years. The veterans in the CON group identified themselves as having various mental health issues (*n* = 1), or physical (*n* = 10), hearing (*n* = 2), visual (*n* = 3), or cognitive impairments (*n* = 1), or other injuries (*n* = 3); and reported that they had their injury/impairment for an average of 8.1 ± 7.2 years. CON participants represented ten sports (e.g., Archery, Cycling) with some having never previously competed in their sport (*n* = 3), whereas others had competed for 6 months to 45 years (*M*_*years*_ = 6.5 ± 13.3 years), at standards ranging from club to international level. The comparison of the IG group and a relatively matched CON group affords an insight into the differences in the stress process encountered by those competing at an international sporting competition and those who are not. Additionally, usage of a control group means that the predominant factor for comparison when examining psychosocial and biomarker measures is the engagement with the IG, rather than alternative confounding factors.

### Procedure

Following institutional ethical approval, military veterans who had been selected for the United Kingdom 2016 IG team were contacted by email about the study, as were military veterans who had not been selected (for the CON group). All veterans who expressed an interest in participating were contacted with further information before providing informed consent. Both groups were asked to complete questionnaires over seven time-points, which reflected competition milestones (e.g., post-selection at 6 weeks before Games, training camps at 1 and 3 weeks before the Games), with pre-competition time-points subsequently mirrored post-Games (e.g., final study time-points were 6 weeks, 3 weeks, and 1 week before the Games; during the Games; and 1 week, 3 weeks, and 6 weeks after the Games). Each questionnaire was selected for its use in previous transactional stress in sport research, and in total took approximately 30–40 min to complete. Data collection predominantly took place online, but paper questionnaires were available on request. The questionnaire packs at the first four time-points contained all seven psychological variable questionnaires detailed below, whilst the questionnaire packs at the remaining three time-points contained six questionnaires as the study only intended to examine the appraisals of stressors in the build up to and at the Games (i.e., not post the Games). Saliva samples were collected (30 min after waking and 30 min prior to sleep to rule out potential confounding by diurnal variation in biomarkers) 1 week before the Games; 24 h after landing in the United States (where the Games were held), 24 h before the first competitive event, and 1 week after the Games.

### Measures

#### Stressors

The 23-item Organizational Stressor Indicator for Sports Performers (OSI-SP; Arnold et al., 2013) measured the organizational stressors that participants encountered during their participation in competitive sport over the past month. The five subscales on the OSI-SP are goals and development (six items; e.g., “my goals”), logistics and operations (nine items; e.g., “the training or competition venue”), team and culture (four items; e.g., “my teammates’ attitudes”), coaching (two items; e.g., “my coach’s personality”), and selection (two items; e.g., “how my team is selected”). For all items at all time points, the stem “In the past week, I have experienced pressures associated with…” was provided, to which the participants responded on three rating scales: frequency (“how often did this pressure place a demand on you?”; 0 = *never* to 5 = *always*), intensity (“how demanding was this pressure?”; 0 = *no demand* to 5 = *very high*), and duration (“how long did this pressure place a demand on you for?”; 0 = *no time* to 5 = *a very long time*). Organizational stressors were measured post-IG as athletes were still associated with HfHSR, who were still offering support to them. Arnold et al. (2013) have provided evidence to support the indicator’s validity and reliability, with acceptable alpha values evident in the present study (α range = 0.76 to 0.91).

#### Appraisals

The 28-item Stress Appraisal Measure (SAM; Peacock and Wong, 1990) assessed the athletes’ primary and secondary appraisals of the stressors they encountered at the time-points prior to the IG. Specifically, the SAM measures primary appraisals (threat, challenge, and centrality) and secondary appraisal (controllable-by self, by-others, and uncontrollable by anyone). For all items (e.g., “Does this situation make me feel anxious?”), participants were asked to respond in accordance with how they viewed the stressors at that moment in time, with all items rated on a five-point scale ranging from 1 (*not at all*) to 5 (*extremely*). Peacock and Wong (1990) reported acceptable reliability for the SAM, which was supported in this study (α range = 0.76 to 0.93).

#### Coping

Coping was assessed using the Modified COPE (MCOPE; Crocker and Graham, 1995). On this measure, 12 coping strategies are presented and participants were asked to indicate, on a five-point scale ranging from 1 (*not at all*) to 5 (*very much*), how much they used each strategy to cope with the pressures they experienced as part of their involvement in competitive sport over the past week. The strategies measured are classified into the higher-order functions of coping, with five categorized as problem-focused coping (e.g., item: “I work harder”), five as emotion-focused coping (e.g., item: “I talk about my feelings with someone”), and two as avoidance coping (e.g., item: “I act as though I am not having pressures”). Crocker and Graham (1995) reported that the alpha coefficients ranged from 0.62 to 0.92 and acceptable reliability was shown in the present study (α = 0.73 to 0.96).

#### Emotions

The 22-item Sport Emotion Questionnaire (SEQ; Jones et al., 2005) measured five emotions: anxiety (five items: nervous, anxious, tense, apprehensive, and uneasy), dejection (five items: unhappy, sad, upset, dejected, and disappointed), anger (four items: annoyed, irritated, furious, and angry), excitement (four items: enthusiastic, excited, energetic, and exhilarated), and happiness (four items: joyful, pleased, cheerful, and happy). Participants were asked to score the occurrence of items in relation to their involvement in competitive sport over the previous week on a five-point scale ranging from 0 (*not at all*) to 4 (*extremely*). The SEQ has been validated for usage of recalling retrospective emotions (cf. Arnold and Fletcher, 2015) as well as its original validation for pre-competition emotions. Jones et al. (2005) reported alpha coefficients ranging from 0.81 to 0.88 and excellent reliability was found in this study (α = 0.90 to 0.95).

#### Well-Being, Health, and Performance Outcomes

Notwithstanding the numerous available measures of well-being (Lundqvist, 2011), the 14-item Warwick-Edinburgh Mental Well-being Scale (WEMWBS; Tennant et al., 2007) was used to measure hedonic and eudaimonic aspects of psychological-well being, including positive affect, interpersonal relationships, and positive functioning. Reflecting on the previous week, participants were asked to respond to items (e.g., “I’ve been feeling useful”) on a five-point scale ranging from 1 (*none of the time*) to 5 (*all of the time*). Tennant et al. (2007) reported excellent reliability for the WEMWBS (α = 0.91), as did this study (α = 0.95). The United Kingdom Short Form 12 Health Survey (United Kingdom SF-12; Ware et al., 1995) was used to measure components of mental health. The questionnaire asked participants to reflect back on the previous week, and a score of α = 0.76 showed acceptable internal consistency. Participants subjectively rated their athletic performance (cf. Pensgaard and Duda, 2003) by responding to the question “Please rate how well you consider your sport performance (including training and competitions) to have been over the past week” on a scale ranging from 0 (*very poor*) to 10 (*excellent*).

#### Salivary S-IgA and Cortisol

Participants were asked to refrain from eating, drinking, smoking, or brushing their teeth for 1 h prior to saliva sampling and to abstain from caffeine and alcohol for 24 h. To rule out potential confounding by diurnal variation in S-IgA and cortisol, participants were encouraged to collect saliva at roughly the same time in the morning and evening on each collection day (Gleeson et al., 2011). Furthermore, all participants were asked to declare all medication, including contraceptive pill usage. In view of the identified impact of exercise on cortisol responses (cf. Jacks et al., 2002), participants’ physical activity patterns were measured using the Leisure Time Exercise Questionnaire (Godin and Shephard, 1985). Objective measures of physical activity (e.g., accelerometers) were not taken, given that participants were locked in to a similar defined behavioral pattern, which was largely determined by preparation for, travel to and performance at the Games. In view of this, it was deemed that there was minimal variability between participants that could have impacted on cortisol concentrations. Unstimulated saliva samples were collected over a 4-min period using the passive unstimulated drool/spitting method (Navazesh, 1993). Specifically, participants were asked to collect saliva on the floor of the mouth without stimulation by orofacial movement or swallowing before drooling/spitting into pre-weighed 15 mL centrifuge tubes at approximately 30-s intervals. Samples were stored at 4°C for up to 24 h, before being weighed to assess sample volume, and centrifuged for 10 min at 1500 × *g* to remove particulate matter. The supernatant was aliquoted, packaged, and transported back to the United Kingdom using dry ice. Samples remained frozen upon arrival and were stored at −80°C until analysis.

Secretory immunoglobulin-A and cortisol were measured using commercially available enzyme-linked immuno-sorbent assay (ELISA) kits according to the manufacturer’s instructions (Salimetrics, Philadelphia, PA, United States). The inter- and intra-assay coefficients of variation for S-IgA were 14.96 and 5.64%, respectively. The inter- and intra-assay coefficients of variation for salivary cortisol were 3.79 and 5.12%, respectively. Average S-IgA secretion rate (μg/mL/min) and salivary cortisol concentrations (μg/mL) were calculated for each saliva sample. In addition to both of these measures and in line with research on diurnal rhythm (Li and Gleeson, 2004), secretion of S-IgA (μg/mL/min∼15 h) and exposure to cortisol (μg/mL∼15 h) over the course of the day was calculated by quantifying the area under the curve (AUCg) using the trapezoid method with respect to ground (Pruessner et al., 2003).

#### Symptoms of Upper Respiratory Tract Infections

Participants were required to complete a daily log in which they documented whether they felt they were suffering from a common cold or flu, and any signs/symptoms of URTI (e.g., sneezing, headache, and malaise). Participants were also instructed to code the severity of the symptom on a four-point scale from 0 (*none at all*) to 3 (*severe*). In accordance with the Jackson Score Questionnaire (Jackson et al., 1958), to be classified as a URTI, the symptoms had to last two or more days, and score greater than 14 (Predy et al., 2005).

### Data Analyses

Using MPlus version 7.4 (Muthén and Muthén, 1998/2015), two-piece linear growth models [in a structural equation modeling (SEM) framework] were used to subdivide measurements into two meaningful time-periods: pre-Games (including competition) and post-Games (including competition). The flexibility of the piecewise growth model allows for the analyses of two distinct time-periods within a longer overall time-frame, without having to conform to assumptions that individual change follows a simple linear trend over the whole time-frame (Preacher et al., 2008). A SEM approach was adopted as it incorporates the observed repeated measures as multiple indicators in one or more latent factors to characterize the unobserved growth trajectories (cf. Curran et al., 2010). Time was centered on the competition time point [i.e., this time-point was labeled zero, with negative (pre-Games) and positive (post-Games) values the further from competition in either direction]; therefore the intercept represented the average score for the variable on the day of competition and the slopes represented the rate of change in the study variables before or after the competition. The intercept and slope coefficients were explored to establish the extent of between-person variation in the intercept and rates of change for all psychological variables (cf. Preacher et al., 2008). Following this, conditional latent growth models (LGMs) were used to determine whether between-person variation in the intercept or slope parameters could be predicted by IG attendance. To elaborate, the time-invariant covariate (TIC) was added to unconditional LGMs as a predictor variable to determine whether any differences existed between the IG and CON groups. Finally, unconditional LGMs with time-varying covariates (TVCs) were used to ascertain whether the veterans’ stress (i.e., organizational stressors, appraisals, emotions) could predict outcome variables (i.e., performance, well-being, and health) at each time point.

## Results

All descriptive statistics for psychosocial measures are presented in Table 1.

**TABLE 1:** Means and standard deviations for variables at all time points for IG group.

**Variable**	**6 weeks prior**		**3 weeks prior**		**1 week prior**		**Invictus Games**		**1 week post**		**3 weeks post**		**6 weeks post**	
							
	***M***	***SD***	***M***	***SD***	***M***	***SD***	***M***	***SD***	***M***	***SD***	***M***	***SD***	***M***	***SD***
**Organizational Stressors Frequency**	1.46	0.83	1.65	0.74	1.47	0.66	1.61	0.83	1.61	0.75	1.09	0.99	1.14	0.93
Goals and Development Frequency	2.22	1.15	2.49	1.27	2.23	1.32	2.16	1.24	2.06	1.20	1.57	1.87	1.75	1.73
Logistics and Operations Frequency	1.39	0.81	1.56	0.84	1.43	0.73	1.54	0.95	1.56	0.88	1.08	0.96	1.09	0.96
Team and Culture Frequency	1.24	1.22	1.55	0.96	1.52	0.82	1.89	1.34	1.54	0.89	1.03	0.51	0.98	0.51
Coaching Frequency	0.63	0.71	0.88	0.61	0.79	0.32	1.00	1.39	0.98	0.55	0.50	0.40	0.55	0.44
Selection Frequency	1.83	1.99	1.79	1.96	1.39	1.78	1.44	2.23	1.93	1.57	1.25	2.31	1.33	2.22
**Organizational Stressors Intensity**	1.68	1.31	1.85	1.03	1.60	0.94	1.72	0.97	1.85	2.24	1.11	1.11	1.18	1.06
Goals and Development Intensity	2.27	1.36	2.55	1.56	2.24	1.71	2.26	1.51	2.11	1.16	1.69	2.46	1.79	2.10
Logistics and Operations Intensity	1.44	0.97	1.68	1.04	1.53	0.89	1.69	1.06	1.49	0.86	1.03	0.89	1.09	0.98
Team and Culture Intensity	1.45	1.72	1.87	1.26	1.71	1.31	2.01	1.66	1.73	1.21	0.98	0.74	0.98	0.73
Coaching Intensity	1.16	2.21	1.23	1.21	1.09	0.86	1.14	1.49	1.03	0.70	0.38	0.21	0.50	0.35
Selection Intensity	2.05	2.27	1.93	2.52	1.41	1.65	1.50	2.45	1.89	1.74	1.37	2.87	1.42	2.72
**Organizational Stressors Duration**	1.59	1.08	1.71	0.98	1.62	0.79	1.70	0.95	1.77	2.17	1.12	1.17	1.17	1.11
Goals and Development Duration	2.19	1.27	2.42	1.39	2.37	1.35	2.36	1.48	2.06	1.20	1.69	2.49	1.84	2.19
Logistics and Operations Duration	1.47	0.96	1.60	1.02	1.58	0.84	1.69	1.03	1.50	0.91	1.11	1.01	1.12	1.03
Team and Culture Duration	1.34	1.38	1.70	1.32	1.69	0.91	1.95	1.48	1.60	1.03	0.99	0.79	1.00	0.81
Coaching Duration	0.94	1.78	1.11	1.32	1.05	0.95	0.98	0.10	0.94	0.49	0.43	0.31	0.47	0.43
Selection Duration	1.99	2.31	1.70	2.15	1.41	1.65	1.50	2.45	1.89	1.74	1.37	2.87	1.42	2.72
**Appraisals**														
Challenge	4.06	0.82	3.91	0.79	4.04	0.90	3.68	1.29	x	x	x	x	x	x
Threat	1.79	0.63	2.26	1.05	2.02	0.66	2.24	1.37	x	x	x	x	x	x
Centrality	3.24	1.01	2.94	1.42	3.17	1.20	3.24	1.57	x	x	x	x	x	x
Controllable-by-self	3.79	0.80	3.70	0.88	3.67	0.78	3.37	1.33	x	x	x	x	x	x
Controllable-by-others	3.59	1.18	3.76	1.04	3.61	0.95	3.41	1.72	x	x	x	x	x	x
Uncontrollable-by-anyone	1.54	0.45	1.89	1.05	1.59	0.48	1.96	0.83	x	x	x	x	x	x
Stressfulness	2.94	0.74	3.17	0.64	2.93	0.55	3.17	1.09	x	x	x	x	x	x
**Emotions**														
Anger	0.45	0.71	0.78	0.96	0.72	0.82	1.15	1.68	1.12	0.98	0.67	0.45	0.53	0.39
Anxiety	2.02	1.34	2.30	1.21	2.05	0.92	2.15	1.66	1.72	1.20	1.17	0.63	1.03	0.08
Dejection	0.49	0.88	0.62	0.87	0.71	1.17	1.19	2.03	1.28	1.23	0.64	0.27	0.59	0.25
Excitement	2.93	0.99	2.74	0.83	2.68	0.52	2.59	1.73	2.34	0.81	2.28	1.17	2.25	1.22
Happiness	2.81	1.16	2.51	1.13	2.46	0.66	2.57	1.50	2.42	1.02	2.50	1.22	2.47	1.20
**Problem-focused Coping Strategies**	3.46	0.84	3.51	0.72	3.57	0.64	3.65	0.65	3.24	0.60	2.80	0.77	2.89	0.97
SSSIR	3.40	0.86	3.30	0.55	3.36	0.53	3.17	0.61	2.73	0.68	2.45	0.65	2.53	0.75
Planning	3.43	1.33	3.45	1.10	3.68	1.03	3.56	1.22	3.51	0.90	3.05	1.27	3.11	1.62
Increasing Effort	3.85	1.29	4.13	0.69	3.91	0.92	4.06	1.04	3.73	0.84	3.32	1.17	3.35	1.22
Active Coping	3.76	1.23	3.76	1.15	3.80	0.96	3.96	0.98	3.46	0.84	3.35	1.44	3.38	1.49
Suppression of Competing Activities	2.86	1.39	2.91	1.26	3.08	0.68	3.52	0.99	2.76	0.97	1.84	0.64	2.09	1.03
**Emotion-focused Coping Strategies**	2.23	0.45	2.47	0.34	2.45	0.73	2.77	0.80	2.27	0.50	1.91	0.23	1.93	0.27
SSSER	2.19	1.02	2.26	0.61	2.48	1.00	2.56	1.55	2.46	0.60	2.32	0.60	2.23	0.69
Venting Emotions	1.48	0.42	1.67	0.42	1.83	1.02	2.36	1.70	1.96	1.01	1.31	0.07	1.28	0.10
Wishful Thinking	2.51	0.65	2.71	0.79	2.56	0.91	3.08	1.44	2.08	1.06	1.96	0.38	1.90	0.33
Self-blame	2.81	1.55	2.93	1.31	2.74	1.35	2.94	1.70	2.68	1.32	2.23	1.37	2.33	1.54
Humor	2.18	1.08	2.76	1.26	2.65	1.38	2.91	1.45	2.21	1.39	1.73	0.65	1.92	0.92
**Avoidance Coping Strategies**	1.43	0.20	1.50	0.25	1.57	0.53	1.61	0.43	1.62	0.43	1.28	0.09	1.29	0.08
Denial	1.64	0.46	1.81	0.63	1.71	0.62	1.94	0.87	1.84	0.54	1.39	0.27	1.42	0.22
Behavioral Disengagement	1.21	0.24	1.19	0.09	1.43	0.67	1.26	0.40	1.39	0.55	1.17	0.13	1.17	0.14
**Well-being**	45.05	10.06	43.23	11.10	46.25	9.69	43.75	12.58	47.15	8.51	53.17	8.69	51.20	9.11
**Mental Health**	36.23	12.73	35.26	12.51	34.54	7.87	36.02	11.09	38.22	9.13	44.08	3.04	42.10	3.86
**Subjective Performance**	6.35	1.73	6.18	3.74	6.60	2.39	6.38	6.38	6.30	3.71	7.07	3.13	6.97	3.50

*SSSIR, Seeking Social Support for Instrumental Reasons; SSSER, Seeking Social Support for Emotional Reasons; x, data not collected for this variable at these time points as questions were focused on the appraisals in relation to the upcoming Invictus Games.*

### Organizational Stressors

**FIGURE 1:**
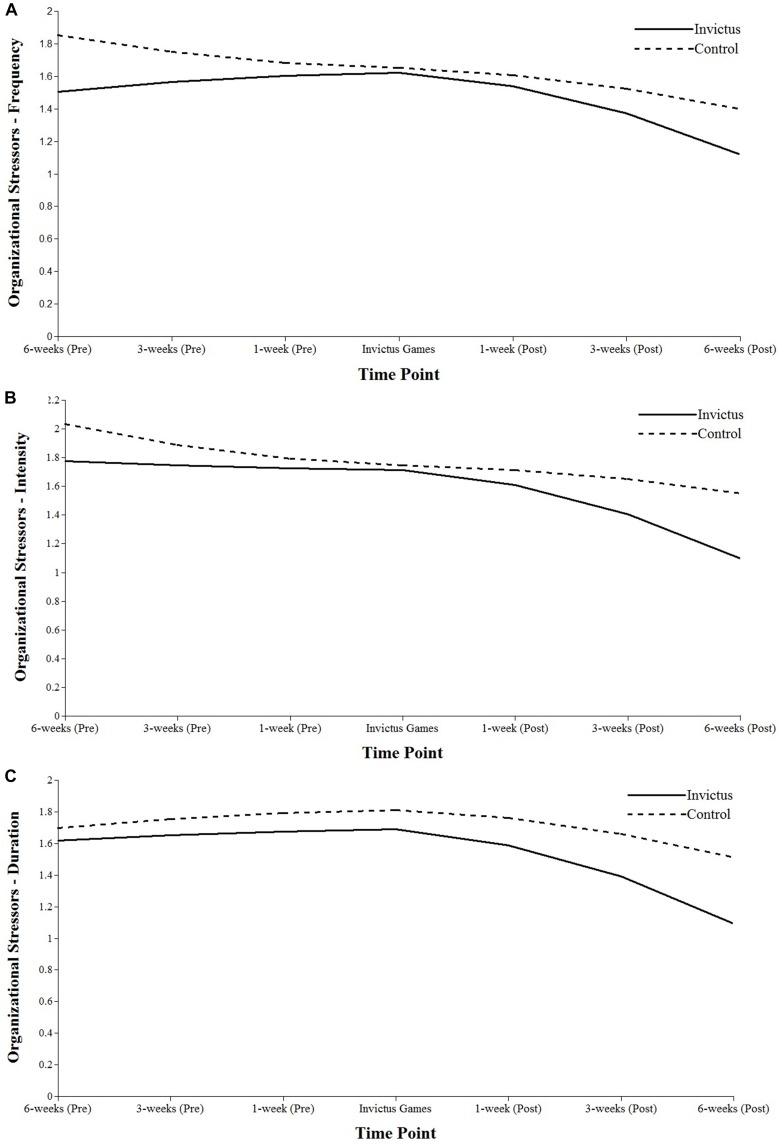
Estimated growth curves for organizational stressor dimensions of frequency **(A)**, intensity **(B)**, and duration **(C)**. Athletes are split into the IG Group or CON Group.

A significant increase (*p* < 0.05) was found in the frequency of team and culture stressors in the build-up to competition. The inter-individual variances in the intercept terms were shown to be significant for the majority of organizational stressors. From competition to the final, post-competition time-point, the frequency and intensity of team and culture stressors (frequency, *p* < 0.01; intensity, *p* < 0.05), and intensity of coaching stressors (*p* < 0.05) all significantly decreased. Including group as a TIC of organizational stressors (see Figure 1) revealed no significant influence at the intercept, although the IG group reported more intense team and culture stressors in the build-up to competition than those in the CON group (*p* < 0.05). Post-Games, the CON group reported significantly higher dimensions of team and culture stressors (frequency, *p* < 0.05; intensity, *p* < 0.01; duration, *p* < 0.05) as well as more intense logistics and operations stressors (*p* < 0.05). The inclusion of organizational stressors as a TVC in the well-being LGM revealed that the three dimensions of these demands (e.g., frequency, intensity, and duration) were negatively related to well-being at each time point (*p* < 0.05). There were significant negative effects of frequency and intensity of organizational stressors on performance for the day of competition (*p* < 0.05). Furthermore, all three dimensions of organizational stressors were negatively related to mental health at each time point in the build up to and at the Games (*p* < 0.05).

### Appraisals

A significant decrease was found in challenge appraisals in the build-up to the Games (*p* < 0.05). Between-person variance was significant at the intercept for the appraisal of centrality (*p* < 0.05), and stressfulness (*p* < 0.01). Including group as a TIC of appraisals showed that in the build-up to competition, IG group participants appraised stressors encountered as uncontrollable-by-anyone more than those in the CON group (*p* < 0.001). Moreover, the CON group made challenge (*p* < 0.01) and centrality (*p* < 0.05) appraisals significantly more than the IG group at the competition time-point. The inclusion of appraisals as a TVC in a well-being LGM revealed that challenge appraisals were positively related to well-being at each time point in the build-up to and at the Games (*p* < 0.01), whilst threat appraisals were negatively related to well-being at each of the same time points (*p* < 0.001). The inclusion of appraisals as a TVC in a subjective performance LGM revealed that threat appraisals were negatively related to subjective performance at time points in the build up to and at the Games (*p* < 0.05). Threat appraisals were negatively related to mental health at all time points in the build up to the Games (*p* < 0.001).

### Coping Strategies

Significant rates of growth (between time-points) were found for the use of seeking social support for emotional reasons (*p* < 0.05), suppression of competing activities (*p* < 0.05), venting of emotions (*p* < 0.001), and humor (*p* < 0.001) in the build-up to the Games. Between-person variance of the intercept terms were shown to be significant for the coping strategies of self-blame (*p* < 0.05), humor (*p* < 0.01), denial (*p* < 0.01), and wishful thinking (*p* < 0.05). Significant decreases in the use of humor and denial were found post-Games (*p* < 0.05). Including group as a TIC of coping strategies showed that in the build-up to the Games, the IG group employed the venting emotions coping strategy significantly more than the CON group (*p* < 0.01). At the competition time-point, the IG group used suppression of competing activities and increased effort coping strategies significantly more than the CON group (*p* < 0.01); whereas those in the CON group used behavioral disengagement coping strategies significantly more than the IG group at this time-point (*p* < 0.01). When coping strategies were included as a TVC in a well-being LGM, problem-focused strategies were positively related to well-being at time-points 2 and 3 (*p* < 0.05). In contrast, emotion-focused and avoidance strategies were negatively related to well-being from time-point 3 to 7 (*p* < 0.01). Avoidance strategies were negatively related to mental health in the build up to and at the competition (*p* < 0.001).

### Emotions

Anger and dejection showed significant rates of growth prior to competition (*p* < 0.001), whereas excitement and happiness decreased (*p* < 0.05). Between-person variance of the intercept terms were shown to be significant for anxiety (*p* < 0.01) and anger (*p* < 0.05). In the post-competition period, anxiety decreased (*p* < 0.05). Including group as a TIC of emotions showed that in the build-up to the Games, the IG group reported anger (*p* < 0.05) and dejection (*p* < 0.01) significantly more than the CON group. Furthermore, at the competition time-point the IG group were significantly more anxious than the CON group (*p* < 0.05). The inclusion of emotions as a TVC in a well-being LGM showed that anger, anxiety, and dejection were negatively related to well-being in the build up to and at the Games (*p* < 0.01). Conversely, happiness and excitement were positively related to well-being at the same time points as well as post-competition (*p* < 0.01). The inclusion of emotions as a TVC in a performance LGM showed that anxiety and dejection were negatively related to performance in the build up to the Games (*p* < 0.05), whereas happiness was positively related to performance in the pre-competition period (*p* < 0.05). Anger, anxiety, and dejection were negatively related to mental health at each time-point in the build up to and at the Games (*p* < 0.05), whilst excitement and happiness were positively related to mental health (*p* < 0.05).

### Salivary S-IgA and Cortisol

**FIGURE 2:**
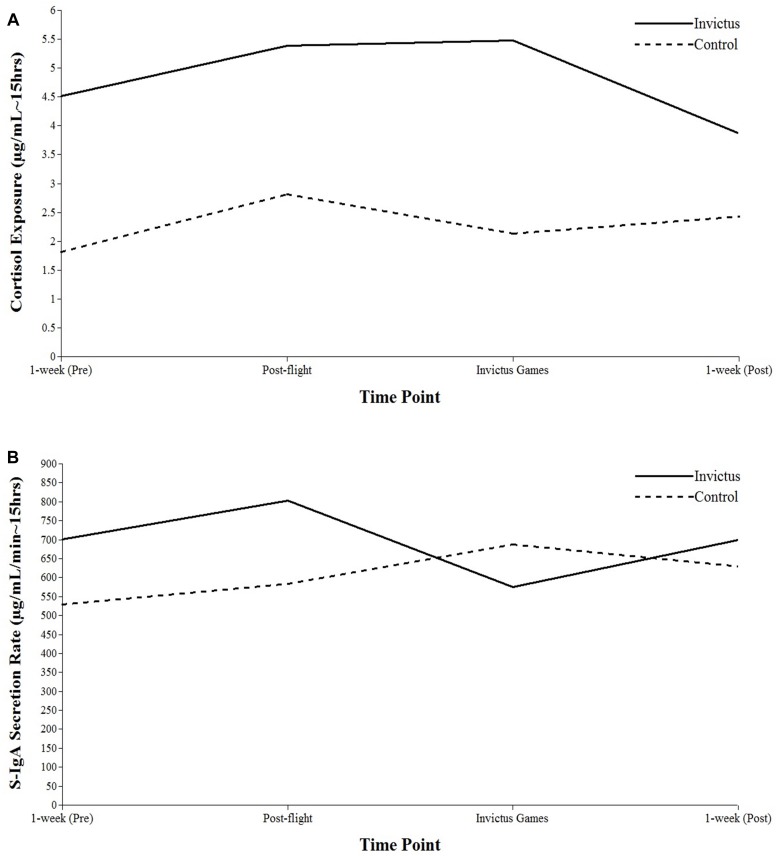
Growth curves for biomarkers of stress: cortisol exposure **(A)** and salivary immunoglobulin A **(B)**. Athletes are split into the IG Group or CON Group.

Table 2 displays descriptive statistics for salivary S-IgA and cortisol. There were no significant changes over time for secretion of S-IgA. Mean scores and between-person variance of the intercept terms were shown to be significant, indicating that S-IgA secretion on the day of competition varied across participants. The inclusion of group as a TIC revealed no significant differences between the IG and CON groups in S-IgA secretion rate (see Figure 2). The inclusion of all psychosocial variables as separate TVCs in S-IgA exposure across the day LGMs revealed no significant findings. There was a significant decrease in cortisol exposure over the course of the study period (*p* < 0.05). Between-person variance of the slope term was shown to be significant for cortisol exposure (*p* < 0.05) meaning that individuals varied in levels of cortisol throughout the study period. Including group as a TIC revealed no significant differences between the IG and CON groups in cortisol exposure (see Figure 2). The inclusion of organizational stressor dimensions as a TVC in the cortisol exposure across the day LGM revealed that intensity of organizational stressors was positively related to cortisol exposure on the day of competition (*p* < 0.05). The inclusion of all other stress variables as TVCs revealed non-significant findings.

**TABLE 2:** Means and standard deviations for biomarkers of stress at all time points for all participants.

**Variable**	**1 week prior**		**Post-flight**		**Invictus Games**		**1 week post**	
				
	***M***	***SD***	***M***	***SD***	***M***	***SD***	***M***	***SD***
**IG Group**								
S-IgA Secretion Rate (μg/mL/min∼15 h)	715.97	824.32	830.53	804.61	595.16	500.61	707.56	518.62
Cortisol Concentration (μg/mL∼15 h)	4.26	6.21	5.44	5.53	5.63	6.31	2.41	1.37
URTI symptom severity	0.89	2.01	0.94	2.15	0.38	1.01	0.35	1.03
URTI symptom duration (days)	0.15	0.36	1.00	1.98	0.58	1.50	0.44	0.86
**CON Group**								
S-IgA Secretion Rate (μg/mL/min∼15 h)	529.12	207.69	583.54	318.09	687.16	452.66	628.74	330.21
Cortisol Concentration (μg/mL)	1.81	0.63	2.81	1.85	2.13	1.21	2.42	1.18
URTI symptom severity	0.00	0.00	0.00	0.00	0.00	0.00	0.00	0.00
URTI symptom duration (days)	0.00	0.00	0.00	0.00	0.00	0.00	0.00	0.00

*S-IgA Secretion Rate (μg/mL/min∼15 h) refers to S-IgA exposure across the day and was calculated using AUCg; Cortisol concentration (μg/mL/min∼15 h) refers to cortisol exposure across the day and was calculated using AUCg; URTI symptom severity was calculated using total symptom scores across the study period and averaged across participants; URTI symptom duration was calculated using total days reported across the study period and averaged across participants.*

### Upper Respiratory Tract Infections

In line with the first criterion of classifying URTIs, that symptoms had to last two or more days and score greater than 14 (Predy et al., 2005), only one participant at the IG reported a cold over a period of 2 days. Using the second criterion of subjective self-report of a cold, only four participants at the IG reported a cold over an average of seven and a half days. Due to a small number of participants reporting URTIs, no further analyses were undertaken.

## Discussion

Research to date has not examined military veterans’ holistic experiences of high-level sport and the antecedents to both positive and negative outcomes. Therefore, the primary purpose of this study was to examine the stress experiences of competitors in preparation for, during, and following the IG and how these experiences may alter over time and in comparison to a control group not participating in high-level competition. Furthermore, a secondary purpose of the study was to examine the relationships between veterans’ stress (i.e., stressors, responses) and psychological, behavioral, and physiological (i.e., salivary measurements of immune and endocrine function) outcomes. The results provide the first longitudinal insight into how the dynamic stress process variables (e.g., stressors, appraisals, coping, and emotions) change over the extended period of a competitive sporting event and, importantly, the relationship between stress and military veterans’ performance, well-being, and health. Contextualized within extant sport psychology literature and theory, these findings are in accordance with the transactional stress process (cf. Lazarus and Folkman, 1984) and can advance extant knowledge and understanding by advancing methodology from cross-sectional snapshots of stress-related variables to more robust examinations of how they can fluctuate over time and predict important outcomes.

This study showed that the organizational stressors encountered by IG participants changed over time. Specifically, team and culture stressors significantly increased in frequency in the build-up to the Games, before decreasing post-competition. These team and culture stressors, which refer to demands associated with attitudes and behavior in the team, may have increased because participants may not have previously encountered these types of stressor in a competitive sporting environment. Indeed, veterans may have reported more of these stressors due to either the new responsibilities they felt they had on their new team, or perhaps certain teammate attitudes that they may not have encountered previously. Furthermore, the increase in frequency and intensity of team and culture stressors may be explained by the increased amount of time athletes spent together when training and preparing for competition. Previous research examining military competitions (Sporner et al., 2009) has suggested that veterans look to their peers for support because they share similar experiences; the findings of this study suggest that this may have been difficult for some athletes, particularly if the stressor had been their teammates (cf. Arnold et al., 2018). Previous research has highlighted that the organizational stressors encountered by a sport performer can be associated at one time-point with negative emotions and performance dissatisfaction (Fletcher et al., 2012b; Arnold et al., 2013). Results from this study advance extant knowledge by finding that, over time, the dimensions of organizational stressors negatively relate to well-being, performance, and mental health in IG participants.

Appraisals are instrumental in the stress process and can provide insight into how a performer responds, and subsequently adapts, to stressors in their sport. In contrast to extant literature (Cumming et al., 2017), the present study found that in the build-up to competition challenge appraisals significantly decreased. As organizational stressors (e.g., logistics and operations) typically relate to environmental factors often out of an athlete’s control (i.e., controlled by the coach or organization), it is likely that athletes felt a lack of control and subsequent mastery of events, particularly close to competition which led to reductions in challenge appraisals. In support of the transactional stress approach, threat appraisals were shown to be negatively related to well-being and subjective performance in the build-up to the Games. This negative relationship is reflective of research in sport psychology where, across a number of sports, threat appraisals are considered maladaptive to performance (Nicholls et al., 2011). This study advances extant literature, however, by demonstrating this relationship over time and with a military veteran sample. This longitudinal focus is important as can provide insight into the temporal occurrence of and fluctuations in stressors and appraisals, and the sequence of events in the stress process (i.e., risk factors for particular health, well-being and performance outcomes). A negative relationship was also found between threat appraisals and mental health in the build-up to the Games. Work by Solomon et al. (1988) showed that military veterans who appraised situations as a threat and did not employ appropriate coping strategies had exacerbated PTSD symptoms. Considering the prevalence of PTSD in military veterans and the topicality of military veteran health (Caddick and Smith, 2014; HM Government, 2018), the findings of this study, highlighting the adaptive nature of challenge appraisals and maladaptive nature of threat appraisals for health and wellbeing, provides an important and significant advance for supporting individuals with such illnesses in a veteran population.

In the build-up to the Games, athletes who utilized problem-focused coping strategies reported higher levels of well-being. Nicholls et al. (2012) demonstrated similar findings among a non-military athlete population, who expressed more emotions that are positive when they engaged in problem-focused coping strategies. Conversely, emotion- and avoidance-focused strategies were negatively related to well-being over the same period. These findings support previous literature, which has demonstrated that not dealing with problems can negatively affect an athlete, regardless of age or level (Nicholls et al., 2012). Furthermore, this study advances previous military psychology literature (cf. Barnett et al., 2016) as it illustrates that avoidance-focused strategies adopted by veterans in the build-up to international sporting competitions are negatively related to mental health. This finding also has implications for practitioners encouraging military veterans to use sport as a form of recovery. Specifically, it is suggested that they look to support veterans with the development and implementation of problem-focused coping strategies in response to sporting demands, given the positive relationship found with well-being in this study and the recognized and topical importance of this outcome for military veterans’ recovery journeys (Weir et al., 2017; Caddick and Smith, 2018; HM Government, 2018).

Negative emotions increased in the build-up to the Games and were negatively related to well-being, subjective performance, and mental health. To explain the heightened dejection reported in the build-up to the Games, it is likely that the IG participants lacked experience of international sporting competition; therefore, feelings of deficiency in terms of their performance may have arisen (cf. Jones et al., 2005). Conversely, athletes experiencing higher levels of positive emotions (e.g., happiness, enjoyment) reported higher well-being, subjective performance, and mental health prior to the Games. Positive emotions have been associated with sport and in particular, individuals appraising themselves as making progress toward a goal (Lazarus, 2000). This may explain the findings that positive emotions reported by military veterans were associated with well-being in the build-up to the Games, since participants were working toward the goal of competing at the IG.

The organizational stressor dimension of intensity was shown to be significantly, positively related to cortisol exposure on the day of competition. Previous research has demonstrated increases in salivary cortisol concentration in response to competition (Casto and Edwards, 2016); however, this is the first study in sport to identify organizational stressors as a potential trigger of the salivary cortisol response. Indeed, it could be argued that this increased exposure to cortisol on the day of competition was in response to the intensity of stressors surrounding competition. Arguably, this rise in cortisol on the day of competition may have been a positive, appropriate, and adaptive response to prepare individuals for extreme physical exertions. Nevertheless, salivary cortisol exposure was shown to decrease over the course of the study. This is in contrast to previous research which has demonstrated anticipatory rises in cortisol prior to competition and even greater increases at competition, prior to an expected decrease post-competition (Casto and Edwards, 2016). Furthermore, previous research has shown that under conditions where participants appraise the situation as a threat, cortisol levels rise; though the effect of this physiological response may be moderated by important factors such as individual difference (Meggs et al., 2016). As previously mentioned, however, there was no significant change in threat appraisals observed prior to competition; therefore, although, challenge appraisal significantly decreased in the build-up to competition, it could be suggested that it is a threat appraisal increase (rather than challenge appraisal decrease) which is related to cortisol fluctuations. These relationships, however, require further investigation.

Although no significant changes were observed for S-IgA secretion across each day or between time-points, it is worth noting that S-IgA was, on average across IG participants, produced at the lowest levels on the day of competition. A potential explanation could be that elevated cortisol levels, due to organizational stressor intensity, suppressed S-IgA secretion. Thus, the findings of this study provide further weight to the argument that factors other than exercise *per se* might affect immune function (e.g., psychological stress or international travel; Campbell and Turner, 2018). Although few symptoms of URTIs were reported in the present study, the reduced S-IgA secretion could have potential longer-term implications for IG participants post-competition. For example, aspects of immune function appear to be impaired among individuals who have suffered physical and psychological injuries (Klokker et al., 1998; Neigh and Ali, 2016). Therefore, practitioners could consider monitoring biomarkers of stress, endocrine function, and immune competency, alongside psychological measures, to ensure that fully informed conclusions regarding an athlete’s health and their ability to perform can be made (Taylor et al., 2015).

A key strength of this study is the population examined, since the diversity of previous life experiences and distinctiveness of military veterans who compete in sport can enrich sports psychology research, which has rarely sampled such individuals. The longitudinal study design employed is also a strength, since it has advanced sport psychology research from examining isolated components of the stress process cross-sectionally to a more theoretically informed transactional approach (cf. Lazarus, 2000). Advantages of this approach are that, compared to cross-sectional research designs, it has enabled changes over time to be established, recall bias in participants to be minimized, and stressors and responses (i.e., risk factors) to be related to particular outcomes with specific reference to their presence, timing, and dimensions. Furthermore, measuring salivary biomarkers of stress, endocrine function (i.e., cortisol), and immune competence (i.e., S-IgA) provides a holistic insight into stress in sport; thus, overcoming previously identified limitations of subjective measurements of stress (Arnold and Fletcher, 2012b). The assessment of both S-IgA and cortisol highlights the complex and dynamic response to stress and provides a novel insight into linking these measures to organizational stressors in particular. It must be noted, however, that the assessment of cortisol may have been affected in female athletes due to their menstrual cycle (Maki et al., 2015). The IG structure determined the relative timing of saliva samples, and as such, menstrual cycle effects on cortisol levels is unclear in this study and should be investigated further in the future. Although the sample in this study is a unique population, a limitation of this study was that it did not consider how stress varies as a result of injury characteristics and presence of comorbidities (e.g., PTSD). Examining these distinct characteristics will provide further insight into the specific experiences of military veterans. In line with the previous suggestion, a pertinent future research direction would also be to conduct research with a larger sample size, which will enable demographic and injury differences to be examined. It should also be highlighted that military veterans are predominantly male (Ministry of Defence, 2019) and this is reflected in this study’s gender ratio. With an increasing presence of females in the military and as veterans (Lundberg et al., 2016), scholars are encouraged to examine in future investigations the potential differences between males and females’ experiences of international sporting competition (cf. Lundberg et al., 2016). A further limitation is that the measures used provide snapshots in time of military veterans’ stress and despite providing valuable insight, they do not provide depth and understanding of individuals’ stress experiences. To address this, qualitative research methods would help to provide a deeper understanding of an individual’s stress experience during international sports events.

Previous research has supported the notion that participation in high-level sport provides various benefits for military veterans (Brittain, 2012). Notwithstanding these benefits, the findings of this study have highlighted that elements of the psychological stress process reported in the build up to, during, and post international sports events can have negative consequences for psychological, behavioral, and immunological outcomes and should be carefully managed. Specifically, it is suggested based on this study’s findings, that practitioners consider the development and implementation of stress management interventions which look to either reduce the presence or dimensions of demands (e.g., a primary stress management intervention focused on reducing the dimensions of team and culture stressors) or better support individual responses to them (e.g., a secondary stress management intervention; Cooper, 2015; Arnold et al., 2017). This secondary intervention could support military veterans in making challenge appraisals and reducing threat appraisals of the specific stressors encountered (cf. Jamieson et al., 2018) and enhancing their problem-focused coping by helping to develop strategies that deal with the stressor (e.g., planning, effort, and active coping). Furthermore, from an applied perspective, monitoring of S-IgA and cortisol in saliva as an indicator of immune and endocrine function in athletes could also be conducted to provide further insight into the potential biological consequences of stress. Findings from these biomarkers, alongside the psychosocial measures, imply that a holistic perspective is warranted toward athlete monitoring prior to and during competition as these factors can interact and impact upon an individual’s functioning (see also Taylor et al., 2015).

To conclude, this is the first study to explore military veterans’ holistic stress experiences during a sports competition period, as well as how this differs to veteran athletes who do not experience competition. The novel findings provide important advancements for research in the sports domain regarding fluctuations in components of the stress process over time (e.g., stressors, appraisals, coping, and emotions) and how these are associated with important outcomes (e.g., performance, health, and well-being). The novel, multidisciplinary findings of this study can enhance scholars and practitioners’ awareness of the psychological and biological markers of the stress response, their interactions, and the relationship with the health and well-being of military veterans. Furthermore, the findings provide further weight to the argument that factors other than exercise *per se* might affect immune function (i.e., psychological stress; Campbell and Turner, 2018) and, for the first time in a sports context, have found that organizational stressors are a potential trigger of the salivary cortisol response. By understanding this study’s findings, practitioners and organizations can help to proactively prevent organizational demands and aid individuals in optimally responding and adapting to encounters, which can ultimately, help to mitigate negative outcomes and proliferate the positive outcomes for military veterans participating in competitive sport.

## Data Availability

The datasets generated for this study are available on request to the corresponding author.

## Ethics Statement

This study was carried out in accordance with the APA ethical standards. This study was approved by the REACH ethics committee at the University of Bath. All participants gave written informed consent in accordance with the Declaration of Helsinki, and anonymity and confidentiality were assured.

## Author Contributions

All authors listed have made a substantial, direct and intellectual contribution to the work, and approved it for publication.

## Conflict of Interest Statement

The authors declare that the research was conducted in the absence of any commercial or financial relationships that could be construed as a potential conflict of interest.
